# Decisions about adopting novel COVID‐19 vaccines among White adults in a rural state, USA: A qualitative study

**DOI:** 10.1111/hex.13714

**Published:** 2023-03-02

**Authors:** Mike Kohut, Liz Scharnetzki, Joseph Pajka, Elizabeth A. Jacobs, Kathleen M. Fairfield

**Affiliations:** ^1^ Center for Interdisciplinary Population Health Research (CIPHR) Portland Maine USA; ^2^ Department of Medicine Maine Medical Center Portland Maine USA; ^3^ Present address: Fletcher School of Law and Diplomacy at Tufts University Boston Massachusetts USA

**Keywords:** COVID‐19, qualitative research, risk perception, trust, vaccines

## Abstract

**Purpose:**

Many people, especially in rural areas of the United States, choose not to receive novel COVID‐19 vaccinations despite public health recommendations. Understanding how people describe decisions to get vaccinated or not may help to address hesitancy.

**Methods:**

We conducted semistructured interviews with 17 rural inhabitants of Maine, a sparsely populated state in the northeastern US, about COVID‐19 vaccine decisions during the early rollout (March–May 2021). We used the framework method to compare responses, including between vaccine Adopters and Non‐adopters.

**Findings:**

Adopters framed COVID‐19 as unequivocally dangerous, if not personally, then to other people. Describing their COVID concerns, Adopters emphasized disease morbidities. By contrast, Non‐adopters never mentioned morbidities, referencing instead mortality risk, which they perceived as minimal. Instead of risks associated with the disease, Non‐adopters emphasized risks associated with vaccination. Uncertainty about the vaccine development process, augmented by social media, bolstered concerns about the long‐term unknown risks of vaccines. Vaccine Adopters ultimately described trusting the process, while Non‐adopters expressed distrust.

**Conclusion:**

Many respondents framed their COVID vaccination decision by comparing the risks between the disease and the vaccine. Associating morbidity risks with COVID‐19 diminishes the relevance of vaccine risks, whereas focusing on low perceived mortality risks heightens their relevance. Results could inform efforts to address COVID‐19 vaccine hesitancy in the rural US and elsewhere.

**Patient or Public Contribution:**

Members of Maine rural communities were involved throughout the study. Leaders of community health groups provided feedback on the study design, were actively involved in recruitment, and reviewed findings after analysis. All data produced and used in this study were co‐constructed through the participation of community members with lived experience.

## 1 INTRODUCTION

Following an uneven rollout, COVID‐19 vaccinations have been made available to adults in the United States since May 2021.^1^ The public health benefits of vaccination are clear: vaccinated individuals are less likely to develop serious symptoms or transmit the virus.^2^ Nevertheless, 21.5% of eligible Americans still had not received any doses in July 2022.^3^ While some people face barriers in accessing vaccines,^4^ 16% of US adults consistently report that they will ‘definitely not’ get vaccinated,^5^ a phenomenon known as vaccine hesitancy. The proportion of rural respondents who express no intention to get vaccinated is nearly twice as high.^6^ To promote vaccine adoption, particularly among rural or other medically underserved populations, we must understand vaccination decisions.

Past research on vaccine hesitancy shows that reasons to delay or reject vaccines vary by disease,^7^ but lessons can be drawn. Hesitancy about MMR vaccination has been associated with a general mistrust of profit‐seeking pharmaceutical corporations and the perception that exposure risk for vaccine‐preventable diseases is low.^8^ Similarly, research has shown that parents' hesitancy towards HPV vaccination is rooted in the belief that their child's risk of infection is low since ‘good’ children delay sexual activity.^9^ In both cases, reasons for hesitation are based on risk perceptions: non‐adopters underestimate disease exposure risk and overstate vaccine risks.

Uncertainty is known to skew risk perceptions^10^ and is associated with distrust in risk‐reduction efforts and avoidance of medical decision‐making.^11^, ^12^, ^13^ Given the degree of scientific uncertainty inherent in an emergent public health threat like a pandemic, distrust can be anticipated. Indeed, research on COVID vaccine adoption reports that Adopters have greater trust in the system or are persuaded by others they trust.^14^, ^15^, ^16^ Non‐adopters are exposed to more misinformation^17^ which is linked to concerns vaccines were under‐studied or about side effects.^18^, ^19^

Regardless of associations with trust or uncertainty, many studies have found that COVID‐19 vaccination intent is shaped by personal risk and concern for others.^20^ Aw et al.'s recent review of research on COVID‐19 vaccine adoption in developed countries identified many factors associated with the behavior. Several factors referred to risk perceptions—risk of personally contracting COVID‐19, personal health risk associated with chronic medical conditions and perceived severity of COVID‐19 more generally. Beyond risk, there are associations between vaccine adoption and other health behaviors like influenza vaccination.^21^ Contemporary research in the US reports demographic dimensions as well—Adopters tend to be older, more formally educated and more liberal.^22^

Decisions to adopt or reject novel vaccines are relevant for planning successful public health responses to emerging diseases. Understanding these decisions entails not only identifying factors but also describing how they relate to decision‐making. To clarify these decisions for people in our community, we interviewed rural inhabitants of Maine to collect situated accounts of how they made decisions about adopting novel vaccines 1 year into the COVID‐19 pandemic.

## 2 METHODS

### 2.1 Research design

Qualitative approaches permit in‐depth exploration of how factors contribute to health behaviors at an individual level, as participants can construct accounts in reference to what they believe is relevant.^23^ We used elements of a grounded theory (GT) approach, including flexible sampling strategies informed by ongoing data collection, and the constant comparative method to ground interpretations and findings, while recognizing that data is socially constructed.^24^ Our goal was not to build an inductive theory, so we modified GT, employing the framework method to structure the analysis and facilitate constant comparison.^25^ To understand the spectrum of COVID‐19 vaccine Adoption/Non‐adoption, we sought to interview a purposive sample of people who were and were not vaccinated until reaching thematic saturation for both groups. The study was determined by the Maine Medical Center IRB to be exempt from oversight based on minimal risk to participants.

### 2.2 Recruitment

The research team collaborated with community partners—Healthy Communities Coalition of Greater Franklin County and Pen Bay Community Health and Wellness—in two rural Maine counties to recruit participants when COVID‐19 vaccines had only recently been made available for adult populations. Beginning in March 2021, our partners directly approached adult community members whom they believed could provide informative insights and told them about a study by MaineHealth on COVID‐19 vaccinations, providing instructions to contact the study team to receive an information sheet on the study and schedule an interview. After eight interviews, we had only recruited vaccine Adopters, and we were no longer identifying new themes in that group. To recruit Non‐adopters, we adopted additional strategies. Based on research suggesting political dimensions of hesitancy,^26^ community partners reached out to organizations they felt would have more conservative representation, including churches, veterans' associations and fish and game groups. We also permitted recruitment through other contacts and in surrounding counties. Some people approached for recruitment declined participation, citing fears about expressing their reasons for not vaccinating. Our strategies allowed us to involve four Non‐adopters. We continued recruitment through May 2021 in an effort to recruit more Non‐adopters, but we were unable to reach saturation with that group. We ended recruitment because evolving information about vaccines were changing the context of adoption.

### 2.3 Data collection

Due to COVID‐19 precautions, MK and LS conducted semistructured interviews via phone or Zoom. Interviewers had no previous relationship with the participants. Interviewers identified themselves as researchers affiliated with MaineHealth, explained that the goal of the study was to understand perspectives on COVID‐19 vaccinations, and obtained consent before the interview. Interviewers were themselves vaccine adopters but avoided expressing personal vaccine preferences to participants. Interviews took 15–45 minutes, and were recorded and transcribed. The piloted interview guide included questions about COVID‐19 experiences and concerns, the perceived likeliness of infection, vaccination status and/or intent, reasons for vaccine decisions, sources of trusted information and perceived vaccine acceptance in the community (see Appendix A). While phrasing and depth of probing differed between interviewers, these differences would not have substantially impacted the analysis since equal numbers of Adopters and Non‐adopters were interviewed by each interviewer. Participants received $35 gift cards. We use pseudonyms to maintain individuality while protecting privacy.

### 2.4 Analytical approach

To organize and analyze our qualitative data, we used the framework method.^25^ The method utilizes a spreadsheet or ‘framework’ wherein analytical units (e.g., individual participants or groups) occupy rows, while categories of information or themes occupy columns. Categorical responses are compared across the framework to identify patterns. Our approach was deductive/inductive and reiterative. MK, LS and JP developed an initial framework with categories deduced from research aims and based on the guide: COVID‐19 concern, reported vaccination status, vaccination intention, reasons for vaccination, vaccination concerns and evaluating information sources. Analysts independently coded transcripts, then met to discuss and revise the framework, adding several inductively derived categories: perceived COVID‐19 danger to self and others, weighing risks, trust in authorities and comments on vaccine development. All transcripts were separately coded and entered into the framework table by two analysts, who checked one another's interpretations. Disagreements were discussed until consensus. Based on prominence in the interviews, relatedness to other themes and its relevance to past vaccine research, we focused this analysis on how individuals judged the comparative risks over COVID‐19 and novel vaccines.

## 3 RESULTS

Seventeen people participated. All were White and living in rural counties; the majority were female, college‐educated and politically Independent (Table 1). Thirteen Adopters provided personal decisions to vaccinate. All participants gave concerns about vaccination when asked, though only seven individuals delayed vaccination based on concerns. Abridged data sets generated and analysed for this study are presented in Tables 2, 3, 4.

**Table 1:** Overview of participants.

Pseudonym	Gender	Age	Interviewer		Education	Political party	Intent to vaccinate
Patricia	Female	60 +	LS		Postgraduate	Democratic	Early Adopter
John	Male	60 +	MK		Postgraduate	Independent	Early Adopter
Chris	Male	40–60	MK		Postgraduate	Libertarian	Early Adopter
Sarah	Female	20–40	LS		Bachelor	Independent	Early Adopter
Mary	Female	60 +	LS		Postgraduate	Democratic	Early Adopter
Rebecca	Female	40–60	LS		Postgraduate	Democratic	Early Adopter
Debbie	Female	60 +	MK		Associate	Independent	Early Adopter
Ashley	Female	20–40	MK		Bachelor	Independent	Early Adopter
Jennifer	Female	40–60	LS		Postgraduate	Independent	Early Adopter
Melissa	Female	40–60	MK		Postgraduate	Democratic	Early Adopter
Hannah	Female	20–40	MK		Bachelor	Independent	Delayed Adopter
Angela	Female	40–60	LS		Associate	Republican	Delayed Adopter
Emily	Female	20–40	MK		Bachelor	Democratic	Delayed Adopter
Samantha	Female	20–40	LS		High School	Independent	Uncertain Non‐Adopter
Jacob	Male	20–40	MK		High School	Independent	Uncertain Non‐Adopter
Tammy	Female	40–60	S		High School	Independent	Resolute Non‐adopter
David	Male	40–60	MK		High School	Republican	Resolute Non‐adopter

**Table 2:** Quotes on weighing risks and COVID concerns.

Pseudonym	Weighing Risks	COVID Concern	Type of Concern
Patricia		“I'm certainly concerned and trying to be careful.”	General
John		“…I definitely don't want to be on a respirator.”	Morbidity
Chris	“…unless there was […] like 75% morbidity rate for people who actually got the vaccine as opposed to not getting the vaccine, it was irrelevant.”	“I was concerned enough to get vaccinated when I could.”	General Morbidity
Sarah	“[the vaccine] just kind of puts you down and out for a day, which is much better than the alternative—getting COVID, so…”	“…I'm very careful like if I go into the grocery store, I always wear my mask, always wash my hands.	General
Mary	“…when you compare that to the secondary effects of COVID for those people who survive it, I would not want to be in their place.”		Morbidity
Rebecca	“…the risks associated with the vaccine are minimal compared to the risks of getting COVID…”	“…I have asthma and I would be in a in a pickle and probably hurting…”	Morbidity
Debbie		“COVID is like the flu on steroids to me…”	Morbidity
Ashley	“…I don't want to be the person responsible for spreading it to others, or possibly causing them to die…”		Community Mortality
Jennifer	“… other than immediately dying the minute they stick it in my arm, I'm probably better off than without it.”	“I was really doing okay until they announced the high risk blood type. […] that, for me, just put me over the not doing so well with the worry.”	GeneralMortality
Melissa	“…given how deadly this virus is […] I still think they should do it, even if there might be some adverse effects.”	“It was a really awful illness. I really don't want to get it again because it was so awful.”	MorbidityMortality
Hannah	“…do I want to be somebody […] who may potentially passing it to somebody else, [where] I might not have symptoms, but they might have very severe, if not deadly symptoms?”		Morbidity Community Mortality
Angela	“…when I saw my husband [who had COVID‐19], how sick he was, I'm like, 'just get vaccinated.' I think it's the right thing to do.”		Morbidity
Emily	“…as much fear as I had about what could happen, I feel like if I were to give COVID to someone I care about that would be way worse, so I was just like, 'Get the vaccine.'”		Community
Samantha	“I think eventually everyone should [vaccinate]. But right now, I get older people getting it because they're in a high risk, where I'm young and healthy. The kids are young and healthy. So…”		Community
Jacob	“I figure, with the shot itself, that really reduces my risk of being able to catch it. Now obviously, it doesn't mean I can can't get it, it just puts you at a lower risk.”	“The only thing I worry about is being […] asymptomatic and passing it on to my parents.” “I have a hard time believing that all these people died strictly due to COVID. […] it's easier to just chalk it up as COVID than it is to actually do the autopsy, find out exactly why they died.”	Community Mortality (minimized)
Tammy	“Why would I risk my health with something unknown like the COVID shot?”	“It just grieves me terribly to think that to get an education in this country nowadays you have to jump through all these hoops that the government is putting up.”	Political
David	“…[vaccination] is like jumping out of a perfectly good airplane.”	“I think [COVID] is how they will systematically get people to fall in line.”	Political

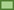
 Early Adopter, 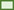 Delayed Adopter, 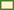 Uncertain Non‐adopter, 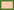 Resolute Non‐adopter

**Table 3:** Quotes on perceived risks of vaccines.

Pseudonym	Common "Reactions": Minimized	Chronic Risks: Specific/Unknown
Patricia	“…no one I've spoken with had any reaction to the first nor did I.”	
John	“*[Anything that concerned you?]* Not really.”	“…just seeing things about pregnant women getting vaccinated and not sure…”
Chris	“I wasn't too concerned about it…”	“…they're not sure what it's going to do to reproduction.”
Sarah	“[after the second dose, my husband] was out for like the day; he didn't feel well.”	“I'd heard something about […] having issues with pregnancy…”
Mary	“…The second shot, some people were laid low for 24 hours […] And others, my husband's, nothing. He didn't have any reaction at all.”	
Rebecca	“The symptoms that I've seen, including my husband on a second shot are so minor…”	
Jennifer	“…next year I might do a little more research, like who had more side effects or I might just stick with one I had, because it wasn't bad.”	
Melissa	“…people were posting what vaccine they had gotten, what side effects they had gotten. […] All this data that people were self‐reporting, and I read all that.”	
Hannah	“The people that I know have said, ‘Oh, my arm really wasn't even sore. I was just tired with the first one and then exhausted with the second one.' And that's really been the only thing.”	“…one of my siblings and his wife are not […] planning to get it. […] they just had their baby a few days ago. And so before when she was pregnant, there weren't any studies yet released.”
Angela		“…maybe long‐term side effects, I was a little bit concerned about. What's going to happen? I don't know…”
Emily	“The doctors that I work with were saying that if you're having a reaction […] within the first few days of getting it then that's a good sign; it means your body's reacting to it and immunities are building up.”	“I felt like there wasn't a whole lot of time to research, like long term effects of it and I felt like there was just a lot of stuff we didn't know about it…”

Pseudonym	*Common “Reactions”*: *Minimized* */* *Emphasized*	Chronic Risks: Specific/Unknown
Samantha	*[What have others told you about their experience getting vaccinated?]* “The first one wasn't too bad. The second one was painful for just a day or two.”	“It's more I just worry about long‐term like, like the blood clot thing like that happening and then you never know.” “…side effects, not knowing, the unknown, really.”
Jacob	“I've heard people have had reactions to it […] but it's very minimal. […] I don't really anticipate having any [allergic reaction], so I'm not really that worried about it, no.”	“…with any vaccine that's rushed the way this has been, I feel like there's going to be some kind of major impact down the road that they haven't become aware of yet.”
Tammy		“…it's so new and there's so little known about it. […] How can you trust it if you don't know what it's going to do to you?”
David	“…that guy ended up getting pretty sick for like two weeks. Then this girl I know, she took the one where you had to take the two doses, and she was fine with the first one, but when she came back for the second one, she ended up getting sick for a while.”	

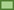
 Early Adopter, 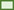 Delayed Adopter, 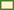 Uncertain Non‐adopter, 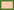 Resolute Non‐adopter

**Table 4:** Quotes on trusting or distrusting the process of vaccine development.

Pseudonym	Trust/Distrust	Development Process: Concerning
John	“I have to trust in the research that was done…”	
Sarah		“…everyone was like, “how can this possibly be? It takes years to create a vaccine and in a short period of time…”
Mary	“I have a background in science […] so I trust and I've done extensive reading. And I trust the vaccines.”	“…since it's so new, I needed to know more about it. […] that it had gone through established trials and that those trials were not somehow rushed.”
Rebecca	“I am not one who believes that news is fake so I trust the institution of journalism, or at least the sources that I look to.”	“Initially I was concerned with the speed with which they were being rolled out, but I have been following enough to say, 'I'm comfortable with it…'”
Debbie	“…it's out of my hands. I'm just gonna have to trust that they're doing it right…”	“It was a concern for me, I always wonder if they go through all the right steps…”
Ashley	“I put a lot of my faith and trust in people who know what they're talking about when I can […] if they know what they're doing, I'm content.”	“…the unknown is obviously very scary. I think that's really it just the lack of data around it. But the more I researched and the more people I spoke to the more confident I felt.”
Jennifer	“…there was a leap of faith in a med being developed in a year that would normally take 10, 15 [years][…] I don't have a lot of trust in the pharmaceutical companies anyways […] they're doing it for profits.”	“…this is a non‐approved med that hasn't gone through rigorous and years' worth of testing, but to kind of read that this is a non‐approved med, was like, 'Oh crap, they don't know. They don't really know.'”
Melissa	“…I guess I trust scientists…”	
Hannah	“I trust that the providers aren't going to give us a vaccine that hasn't been properly studied…”	“One of the biggest things was thinking that it wasn't studied or the vaccine was rushed to be put together…”
Angela	“I mean, it was very quickly done, but, I mean, we have to trust the science on that.”	“I'm certainly not anti‐vaccine, […] [but] I'm not also 100% certain. I feel like we're in an experiment and we'll find out years down the road, how this all plays out. So do I want to be part of that?”
Emily	“I just have to trust in the scientists and the people that have created and tested these vaccines […] I hope that they wouldn't release it to the public if there was seriously something bad that could happen […] you know, blind faith, I guess.”	“Probably because I had the same fears as everyone else where, you know, it just it came on really quickly, and I felt like there wasn't a whole lot of time to research like long‐term effects of it and I felt like there was just a lot of stuff we didn't know about it.”
Samantha		“It's so new, and side effects and everything don't know really too much about long‐term effects of it and all that.”
Jacob	*[What type of person would you trust?]* “Definitely not a politician. It's not the President. No one in Senate or Congress. Even some of the doctors, I feel, are kind of biased on it. They don't want to do it, but they're kind of obligated to suggest it.”	“…the data is not there. […] I get why they're trying to put them out so quickly. But you don't have any historical data to really go from. Right now, everyone that's getting this vaccine and some of this, I kind of feel like they're the sheep, they're the lab rat.”
Tammy	“I find it very, very difficult to trust and believe anybody at this point. I'm just having difficulty with that in general. So as much as I'd like to say I trust my healthcare provider, not really.”	“…it has not been approved by the FDA. It's still in the trial stage. It hasn't been proven. So the side effects of it are unknown and I'm not going to be a gosh dang Guinea pig for anybody.”
David	“…I just don't believe in some of that stuff.”	

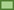
 Early Adopter, 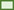 Delayed Adopter, 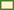 Uncertain Non‐adopter, 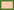 Resolute Non‐adopter

Our results are organized into three subsections. We first present the Spectrum of Hesitance, followed by Weighing Risks. Our third subsection explores social and political contexts for vaccine decisions. Supporting quotes not included in our tables are presented in‐text. They were selected based on clarity and inclusion of voices. For all quotes, brackets indicate added information. Bracketed italics indicate questions from the interviewer, and bracketed ellipses […] indicate deletions to improve clarity.

### 3.1 Spectrum of hesitance

All our participants spoke about vaccination as a personal choice. No participant reported delaying vaccination due to logistical or structural barriers, though one Adopter said convenient locations made vaccination easier.

We distinguished Adopters from Non‐adopters based on whether they had at least scheduled their first shot. We further subdivided Adopters into Early Adopters—who pursued vaccination when available, without intentional delay—and Delayed Adopters who delayed before vaccinating. We subdivided Non‐adopters based on openness to vaccination. Uncertain Non‐adopters were willing to vaccinate but equivocal. For example, Jacob was still considering vaccination at the time of the interview, whereas Samantha was open to vaccinating after ‘a couple of years’. Both thought others should vaccinate, but not them. Resolute Non‐adopters communicated unequivocal opposition to the COVID‐19 vaccines. Table 1 shows participants' assigned categories.

Comparing Adopters and Non‐adopters, several patterns emerge. All participants older than 60 were vaccine Adopters. All Adopters in our sample had at least some college education, whereas Non‐adopters did not. Finally, though most participants identified as politically Independent, all Democrats in our sample were Adopters.

### 3.2 Weighing risks

Most respondents framed their decisions by comparing the perceived risks of COVID‐19 and vaccines (see Table 2). For example, one Resolute Non‐adopter used a metaphor of sky‐diving to convey his risk calculation for vaccination:

I don't want to intentionally inject myself with a virus that I didn't contract on my own. […] it's like jumping out of a perfectly good airplane. […] You're putting your life at risk […] You don't know the idiot that packed that 'chute…. (David)

By contrast, most Adopters asserted that the risks of COVID‐19 were much greater than any vaccine risks:

Other than immediately dying the minute they stick it in my arm, I'm probably better off than without it. (Jennifer, Early Adopter)

The specific risks being weighed—of COVID‐19 and of COVID‐19 vaccinations are presented below.

#### 3.2.1 Perceived risks of COVID‐19

All Adopters agreed COVID‐19 was harmful to health, if not personally, then to the community (Table 2). Many Adopters referenced morbidity risks, emphasizing the possibility of severe acute symptoms from COVID‐19 and long‐term impacts on health, including long COVID and hospitalization. Most Early Adopters were in high‐risk groups, including four participants older than 60 and several others with comorbidities. Some Adopters identifying themselves as ‘low risk’ for COVID severity downplayed personal health risks but instead referenced the psychological harm of being responsible for infecting others:

Seeing firsthand how it impacts a community, that was enough to want to get the COVID vaccine, because I don't want to be the person responsible for spreading it to others, or possibly causing them to die. I mean I just would never be able to live with myself after that. (Ashley)

Non‐adopters never referred to morbidity risks of SARS‐CoV‐2 infection. On the contrary, Tammy drew an analogy from her *actual sickness* from influenza, *not* to COVID‐19, *but instead* to potential side effects from COVID‐19 vaccines:

I mean, I got the flu and I was in bed for three days straight, just out of my gourd with a fever. Just not good at all. So why would I risk it with something? Why would I risk my health with something unknown like the COVID shot? (Tammy)

Rather than mention morbidity or other risks, Non‐adopters focused almost exclusively on mortality risk, which they downplayed. David and Jacob both suggested COVID‐19 was less fatal than authorities claim:

…I have a hard time believing that all these people died strictly due to COVID. I feel like a large majority of them, where they were elderly and things, they probably had underlying conditions…. (Jacob)

While Samantha and Jacob judged their personal risks as low, they acknowledged that COVID‐19 could be dangerous to other people. However, they presented this as a reason to delay vaccination—to allow high‐risk individuals access to limited vaccines:

…I kind of feel like the people that are sick, could be elderly […] they should have first grabs at all. I mean, people like me, my girlfriend, younger people, aren't really at risk of it being fatal. (Jacob)

Several respondents, including some Uncertain Non‐adopters, also referenced the social risks of an ongoing COVID‐19 pandemic. They hoped vaccination would allow a return to normalcy, including gathering socially without masks. Several participants who work with the public cited work‐related benefits of vaccination, such as allowing flexibility with masks:

It's really difficult to say to a family in their own homes, you need to put on a mask in your home, because I'm here. So I have been probably less rigid with that rule than I should be […] So [vaccination] just made sense. (Jennifer)

Participants did not specify beliefs about the degree of immunity conferred by vaccination. Some acknowledged vaccines would not give complete protection, while several Adopters referenced ‘herd immunity’, and suggested vaccines would slow transmission and thereby protect people who could not be vaccinated:

I think that the more people who get it, I think that we will gain the immunity that they're talking about, that so‐called herd immunity. I think the more Americans who are getting this is just going to overall help those who maybe can't get the vaccine. (Hannah)

Responses from all participants were consistent with the belief that the vaccines were, to some extent, protective against COVID‐19. No Non‐adopters disputed this premise within the interview.

#### 3.2.2 Perceived vaccine risks

Several Early Adopters said they never questioned whether to vaccinate, explaining they saw no reason *not* to vaccinate. Nevertheless, all respondents were specifically asked, and most responded with specific risks of vaccinations (Table 3). Participants from all hesitancy groups downplayed common ‘flu‐like’ reactions immediately following vaccination or understood them as evidence vaccines were working (Emily). When Adopters talked about other more serious side effects reported in mainstream news, including blood clots, they accepted them as risks, but vaccinated anyway. Some even invoked them to illustrate the system is effective at identifying harms:

…like what happened with the J&J vaccine when people started getting blood clots: if there was something really bad we would hear about it because people would be reporting it. (Melissa)

By contrast, for Non‐adopters like Samantha, known issues like blood clots were not evidence the system was working, but instead evidence other harms were possible. While some referenced known risks like anaphylaxis or blood clots, all 4 Non‐adopters emphasized possibilities of the unknown, longer‐term side effects (see Table 3). By contrast, only half of the Adopters mentioned other unknown risks, mostly around women's fertility. Despite hearing these side effects, Adopter's comments suggested a lack of concern.

Several Non‐adopters (David, Tammy and Jacob) expressed political concerns about vaccines, especially about *coerced* vaccination, including vaccine requirements for travel or entry:

I think the only thing that worries me is they're going to force me to take a vaccine I don't want. (David)

They expressed such concerns multiple times in the interview without prompting. These concerns were not merely personal, but perceived as harmful to the body politic:

I'm in tears because of how much the thought of giving up our rights […] I think that they are withholding information from us that they already know. I think that's a crime and it scares me to the point where I am actually crying, mourning my grandchildren who aren't even developed mentally to the point where they will stand up and say, ‘No, I will not do this’. (Tammy)

### 3.3 Contexts of decisions

#### 3.3.1 Speed of development and uncertainty

COVID‐19 vaccines were developed and released less than a year after the SARS‐CoV‐2 virus appeared. Most participants (10) commented on this timeline without prompting (see Table 4). This relatively short timetable of development and approval was a source of uncertainty for many participants, though the implications of this uncertainty varied. At the least, several Adopters acknowledged that certain facts about the vaccines, like long‐term efficacy, were unknown. Both Delayed Adopters and Non‐adopters suggested the short timeline was the reason for additional concern—about unknown, long‐term effects:

I'm certainly not anti‐vaccine, I am not. But on the same hand, I'm not also a hundred percent certain. I feel like we're in an experiment and we'll find out years down the road, how this all plays out. So do I want to be part of that? Or how do I want to participate with that? (Angela)

However, Adopters described ultimately deciding to trust the process. Some sought information to address their uncertainty, consulting doctors or doing their own ‘research’.

Non‐adopters drew more elaborate implications from the uncertainty. Jacob and Tammy argued that, because the vaccine was insufficiently tested, people being vaccinated were comparable to ‘lab rats’ or ‘guinea pigs’. Samantha and Tammy considered being vaccinated in the future, but only after the test of time removed uncertainties:

It's so new, and side effects and everything don't know really too much about long‐term effects of it and all that. But after some time, if everything works out good, I'll probably get it. (Samantha)

Tammy suggested considering the vaccines after a decade or more.

#### 3.3.2 Social media amplifying concerns

Participants referenced social and political contexts that shaped how they evaluated information about COVID‐19 and/or vaccine risks. Many participants reported seeing concerning claims about vaccines on social media. Adopters described social media as an information source while acknowledging much was inaccurate. When Adopters encountered concerning claims, they concluded they were not credible:

*[Have you heard anything any like conflicting or contradictory messages about the vaccine?]* Oh yeah. I mean from people that are against getting it obviously. It just all sounds crazy to me like the fact that people think the vaccine has microchips in it? I'm just like what? It doesn't make sense. (Emily)

Even when dubious, some Adopters acknowledged these messages can still be powerful:

…there's a lot of negativity around the vaccine [on social media], saying ‘Oh yeah, they're just going to put a chip in that and they're going to control everybody’. Well, that's not the thing and I obviously knew that that wasn't the case, but at the same time, it's hard to feel positive about something when there's so many negative things. So yeah, I would just try to tune it out. (Hannah)

In contrast, Non‐adopters like Tammy drew stronger inferences after hearing concerning information on social media:

How can you trust it if you don't know what it's going to do to you? I've heard that there is—this was from kind a conspiracy theorist so I kind of take it with a grain of salt—but almost like Sci‐Fi nanobot, bio‐technic stuff in the shot. […] And that makes me very, very uncomfortable that we have so many people pushing this on us. (Tammy)

Despite taking these allegations ‘with a grain of salt’, Tammy nevertheless imputes mal‐intent to vaccine promoters, supported by the claims. Similar conspiratorial claims included that information is hidden from the public, or that SARS‐CoV‐2 was created for political purposes:

…honestly, at this point, is this really happening? Is this natural or did this just happen to get created and released because it's an election year? What's the benefit of lining somebody's pocket? Who's trying to get control? (Jacob)

While we only heard three individuals imputing credibility to these claims, five other participants described having heard similar claims on social media and disregarding them.

#### 3.3.3 Reasons for distrust

Both groups recognized reasons to distrust official recommendations about the vaccines, including changing recommendations on COVID‐19 precautions from public health authorities:

To hear Dr. Fauci say masks don't work and then to hear him say masks do work, that was a problem for me. (John, Adopter)

The reason why I kind of distrust the CDC and ‘experts’ in the field is because they flip flop. First they say, ‘Oh, don't do this. Oh, don't do that. Oh, this is fine. That is fine’. And then they turn around and they say, […] ‘Yeah, we got that wrong’. […] It just makes me very distrustful of them when they flip‐flop like that. (Tammy, Non‐adopter)

The profit motives of pharmaceutical companies were also cause for distrust:

…they haven't studied it. They're more worried about, ‘Well, hey, let's get this out. Let's get some money. Let's do this’. I think the safety is a little bit of a concern, but I think it's a very small percentage when compared to how much medical stocks have raised. (Jacob)

Jennifer (Adopter) echoed this concern, alluding to the ongoing opioid epidemic:

We can't even develop non‐addictive pain medicine, but we could come up with three vaccines for a pandemic? […] I don't have a lot of trust in the pharmaceutical companies anyways. They're not doing it to benefit humans. They're doing it for profits. (Jennifer)

While participants from across the spectrum agreed these issues were reasons to distrust vaccines, they arrived at different conclusions. Jennifer presented good reasons for skepticism, but she described overcoming her distrust:

…there was a leap of faith in a med being developed in a year […] *But the bottom line is hopefully because it's a global pandemic, there is more altruistic motives* than, than financial motives for these companies. (Jennifer)

Fearing they would bring her more uncertainty, the same participant actually chose to avoid reading vaccine warnings:

…when they handed me the info sheet for the first vaccine, I started reading it and then threw it away because if I kept reading it, I was going to walk out and not get the vaccine. (Jennifer)

In contrast, Tammy cited changing recommendations as a reason to distrust the vaccine, which in turn convinced her to distrust doctors recommending the vaccine (see Table 4).

#### 3.3.4 Whom to trust?

The clearest division between Adopters and Non‐adopters was trust in relevant authorities (see Table 4). Nearly every Adopter (10/13) justified vaccination decisions by declaring trust in science. Jennifer, Ashley and Emily used the language of faith to describe their trust in science despite doubts (see Table 4 for supporting quotes). In contrast, no Non‐adopters described trusting scientific or medical authorities. Instead, they sometimes suggested trusting these authorities would lead to harm, comparing people who trust the vaccine with experimental animals.

Adopters also expressed trust in medical providers. Several described overcoming initial concerns after consulting with providers. By contrast, Non‐adopters never described physicians as trustworthy, though Jacob said he planned to discuss it with his doctor during his next visit. Mostly Non‐adopters said they trusted friends and/or family:

Even some of the doctors […] don't want to do it, but they're kind of obligated to suggest it. […] the only people that I'd actually really rely on their opinions are, is my family. Just because Joe Schmo down the road is going to get it, don't mean I want it, don't mean I'm going to listen to his opinion or how he feels about it. But if it's somebody directly in my household, that makes sense, because I live with them. I don't live with that dumb ass down the road. (Jacob)

#### 3.3.5 Personal networks

Many Adopters, including all three Delayed Adopters, cited other people as influential in their decisions. Ashley admitted to some early concern, ‘But the more I researched and the more people I spoke to the more confident I felt’. Sarah and Emily's concerns were allayed by vaccinated co‐workers and other acquaintances who reported positive experiences. After seeing negativity around the vaccines on social media, Hannah decided to schedule an appointment with a provider who answered her concerns about the vaccines. This was instrumental in her vaccination decision. Another Delayed Adopter, Angela, joked that she got vaccinated to avoid being harassed by others. Though she provided other motivations, Angela's testimony suggests a strong role for others in convincing her to vaccinate so she can say she ‘did her part’ to protect the community. Similarly, Jacob claimed he was considering vaccination despite doubts, in part, because his girlfriend was encouraging him.

## 4 DISCUSSION

Despite issues recruiting Non‐adopters, our findings are consistent with previous studies that reported correlations between vaccine hesitancy and lower educational attainment, lower levels of trust in experts and acceptance of misinformation.^14^, ^15^, ^16^, ^17^ Though political affiliation has been implicated with hesitancy in other studies,^22^ the relationship is less clear in our sample. A much clearer connection is evident for education—every adopter had at least some college education, and no Non‐adopter had above high school education. This pattern is documented in much larger US samples^16^, ^27^ and in other countries.^28^, ^29^ This educational difference coincides with distrust of experts both in this study and in others,^15^, ^16^, ^30^ which may reflect attitudes about the value of education credentials, which are extended vicariously to the trustworthiness of those who hold them.^31^, ^32^, ^33^

We are not aware of previous work reporting differences between Adopters and Non‐adopters on the *kinds of risk* considered relevant. While participants in both groups justified COVID‐19 vaccination decisions by comparing disease risk to vaccine risk, Adopters ultimately said that protection against COVID is a great benefit and worth vaccine risks, while Non‐adopters presented vaccine risks as too great for too little benefit. They reached different conclusions because they considered *different kinds* of potential harm in their judgments—Adopters talked about morbidity risk and potential guilt from infecting others. Non‐adopters focused on low perceived mortality risk, without referring to risks of serious illness. Non‐adopters expressed more concern about sociopolitical harms with the COVID pandemic, including loss of rights and coerced vaccination. While Adopters downplayed known vaccine risks, Non‐adopters emphasized the potential for unknown, longer‐term risks. Additional research is needed to determine whether this pattern holds true with larger samples.

This novel finding is relevant to studies of hesitancy more generally. Similar to hesitancy for other vaccines, our Non‐adopters downplayed vaccine benefits by minimizing harms associated with COVID‐19. However, in contrast to measles or HPV vaccines, wherein low‐risk perception reflects perceptions that *infection* is unlikely,^7^, ^9^, ^34^ low‐risk perception for COVID‐19 reflects skepticism about disease *severity*. In this regard, despite the higher lethality of COVID‐19, the closest analogy for understanding COVID‐19 vaccine health behaviors may be flu vaccination. In the United States, these vaccination rates are even lower than for COVID‐19, at 50.2% during the 2021–2022 season.^35^ If vaccine protection against COVID‐19 wanes over time and requires booster vaccines, this comparison to flu vaccination becomes more apt.

Public perception and utilization of information about COVID‐19, particularly as it relates to risk and risk reduction, shapes motivation to adopt preventative measures, including vaccination. Many participants described uncertainty around the COVID vaccine development process, but uncertainty did not necessarily translate to distrust or vaccine refusal, echoing Gillman et al.'s findings from a national survey.^36^ In our study, faced with uncertainty, Adopters emphasized their faith in medical experts who recommended the vaccines, whereas Non‐adopters warned against trusting the pharmaceutical companies that developed the vaccines and anyone else who promoted them. Rather than faith or trust in experts, Gillman et al. suggested that vaccine intent was moderated by each individual's ‘uncertainty tolerance’.^36^

These findings suggest areas to target for public messaging to moderate how people weigh the risks of vaccinations. To the extent individuals focus on rates of mortality, they underestimate other risks of infection to themselves and fellow community members. Emphasizing other possible harms—missing work, physical distress, hospitalization, medical bills and even long‐COVID—could help outweigh concerns around vaccine risks. Furthermore, if faith or uncertainty tolerance moderates health behavior adoption, it is relevant for public health communication strategies to understand the extent to which the ability/willingness to accept uncertainty is malleable or determined by context.

Our findings demonstrate the centrality of trust in medical professionals and institutions for making decisions about novel vaccines. Trust is built or eroded through cumulative experience. At the time of data collection, several pharmaceutical companies, including one involved with vaccines, were successfully avoiding lawsuits for products causing harm.^37^ Many drug developers are alleged to have misled authorities and doctors about the addictiveness of opioids.^38^ People have good reasons to be skeptical,^39^ and so it is remarkable that so many people in rural Maine and elsewhere have chosen to adopt COVID‐19 vaccination. They have made that choice, by their own accounts, because they trust scientific and medical authorities. Without that trust, people rely on their own ‘research’ or rely on friends, family, co‐workers and others they trust. To ensure that more people vaccinate in the future, public health messages aimed to influence vaccine decisions could be coupled with efforts to make the *process* of drug and vaccine development more trustworthy.^40^

### 4.1 Limitations

Our qualitative approach does not allow us to make claims about how common the various motivations are in the population, both because of our sample (purposive and nonrepresentative) and our method of semistructured interviewing, which privileges the discovery and exploration of novel themes over the ability to quantify specific content.^41^

As we did not reach saturation for Non‐adopters, additional concerns are possible. Nevertheless, as noted above, previous work on vaccine hesitancy suggests our Non‐adopters were not anomalous. Because recruitment was based on connections to community health groups, our sample may not represent community members who are unengaged with health care. Finally, the COVID‐19 pandemic has continued to evolve since the completion of interviews, including the Delta and Omicron wave, with increasing breakthrough cases and recommendations for additional booster vaccinations, thus all results should be understood in a temporal context.

## 5 CONCLUSION

Many respondents framed COVID vaccination decisions around comparing risks between the disease and the vaccine. Associating greater risk with COVID‐19 diminishes the relevance of risks from vaccines, whereas skepticism about risks from COVID‐19 heightens their relevance. Our novel findings on how people think differently about COVID‐19 morbidity risk as opposed to mortality risk could be explored in other contexts around the world. These insights could be used to address vaccine hesitancy in the future.

## CONFLICT OF INTEREST STATEMENT

The authors declare no conflicts of interest.

## Supporting information

Supplementary information.

## Data Availability

Abridged data sets generated and analyzed for this study are presented in Tables 2, 3, 4. The full data set may be requested from the corresponding author. Full transcripts are not being made available due to concerns about participant confidentiality.
