# Supplementary material for: Decisions about adopting novel COVID‐19 vaccines among White adults in a rural state, USA: A qualitative study
Source: Health Expect. 2023 Mar 2;26(3):1052–64. doi: 10.1111/hex.13714 (PMC10154856; doi:10.1111/hex.13714)
Supplement: Supplementary file 1 — Supplementary information. [file HEX-26--s001.docx]

**INTRO:** Thank you for taking the time to speak with me. We are interested in your thoughts and opinions about the COVID-19 vaccine so we can better understand what people know about it and what more information they might need. There are no right or wrong answers and you do not have to answer any questions you do not feel comfortable answering. You can also end your participation at any time, without a reason.

Before we begin, I want to remind you that our conversation will be recorded. During this interview please do not provide any personally identifying information about yourself (like your name). We want to protect your privacy.

At the end of at the conversation we will ask you a few brief demographic questions. When we’re done, the research coordinator will mail you a $35 as a thank you for your time.

Do you have any questions? If not, let’s get started.

Questions:

1. How has COVID-19 affected you and/or your family? Could you give me some examples?
   1. Possible probe: Have you or anyone close to you been diagnosed with COVID-19?
      1. If yes, probe 🡪 who/what is your relationship to them?

b. If yes, probe 🡪 Did they have to be hospitalized?

2) Perceived Risk of COVID-19:

- 1. How worried are you about getting COVID?
     1. On a scale of 1 to 10, where 1 is not at all worried and 10 is very worried, how worried are you that you will get COVID-19 at some point?
        1. Probe why that number
  2. How likely do you think it is that you will get COVID?
     1. On a scale of 1 to 10, where 1 is not at all likely and 10 is very likely, how likely do you think it is that you will get COVID-19 at some point?
        1. Probe why that number

3) Have you been offered the ability to get a COVID vaccine?

- 1. *If yes 🡪* Did you take it? Why/Why not
  2. *If no 🡪* If you were to be offered a COVID vaccine in the next 3 months would you take it? Why or why not?
     1. Probe- If you would not take it in the next three months, would you take it a year from now?
  3. What, for you, are some of the reasons for taking/not taking a COVID vaccine?
  4. What, for you, is the most important reason for taking it/ not taking a COVID vaccine?
     1. Do/did you have any concerns about the vaccine?
  5. Does it matter to you which vaccine you get?
     1. If you had the opportunity to pick, would you get a vaccine with two dose or one dose? 🡪why
     2. If you had the opportunity to pick which vaccine you could get (Moderna, Pfizer, J&J), which one would you pick? 🡪 why?

4) Do you know where you could go to get a COVID vaccine? Where?

5) What kinds of things have you heard about the COVID vaccines?

a. What do you think about that?/Do you believe that?

7) Do you think most of your friends/neighbors will be willing to get the vaccine? Why do you think that?

1. Have you talked with anyone else about it recently? What did they say?
2. Why do you think other people might decide to get/not get it?

6) Do you think that other people ought to get the vaccine?

1. What would be the advantage/disadvantage if everyone got it?

8) How important is it for you to know about how the COVID vaccines should work?

a. Can you share with me what you know about how the COVID vaccines work to build immunity to the coronavirus?

9) Where do you typically get your information about the COVID-19 vaccines?

1. Have you seen any conflicting information about the COVID-19 vaccines?
2. How do you deal with any confusion that conflicting information may create?
3. Social media probe

10) Who do you trust to get good, truthful information about the vaccine?

1. Probe specific people or kinds of people (e.g. the governor, Fauci, scientists, your PCP, religious leader, news source, Dr. Shah, etc.)

11) What would help you, your family, or members of the community seriously consider getting vaccinated?

12) What are your thoughts on getting other vaccines (flu, tetanus)?

1. If different from COVID-19 🡪 What is it that makes you think differently?

If not different 🡪 Tell me a little about what you think about getting other vaccines.

Running probe: Can you share with me some of the things you’ve heard about COVID-19?

1. Probe: What do you think about that (idea)?
2. Do you remember where you heard that/where you got this information?

Demographic/Survey items

1. Gender identity
2. Racial identity
3. Ethnic identity
4. Age (in years)
5. Education level (MS or less, less than HS, HS graduate, Some College, College grad)
6. Occupation
7. Zip code
8. Do you identify with a political party?
   1. If yes🡪 Which political party you most affiliate with -Dem, Repub. Dem Socialist, libertarian, independent other

Would you be interested in being re-contacted in the future to assist us with other parts of our study?

If yes 🡪 we will let the research coordinator know and they will reach out to you with next steps

If no 🡪 thank you for your time and participation.
